# Natural Chalcones for the Management of Obesity Disease

**DOI:** 10.3390/ijms242115929

**Published:** 2023-11-03

**Authors:** Maria Maisto, Adua Marzocchi, Niloufar Keivani, Vincenzo Piccolo, Vincenzo Summa, Gian Carlo Tenore

**Affiliations:** Department of Pharmacy, University of Naples Federico II, Via Domenico Montesano, 59, 80131 Naples, Italy; adua.marzocchi@unina.it (A.M.); niloufar.keivani@unina.it (N.K.); vincenzo.piccolo3@unina.it (V.P.); vincenzo.summa@unina.it (V.S.); giancarlo.tenore@unina.it (G.C.T.)

**Keywords:** natural chalcones, anti-obesity, adipocyte differentiation, lipid accumulation, browning process

## Abstract

In the last decade, the incidence of obesity has increased dramatically worldwide, reaching a dangerous pandemic spread. This condition has serious public health implications as it significantly increases the risk of chronic diseases such as type 2 diabetes, fatty liver, hypertension, heart attack, and stroke. The treatment of obesity is therefore the greatest health challenge of our time. Conventional therapeutic treatment of obesity is based on the use of various synthetic molecules belonging to the class of appetite suppressants, lipase inhibitors, hormones, metabolic regulators, and inhibitors of intestinal peptide receptors. The long-term use of these molecules is generally limited by various side effects and tolerance. For this reason, the search for natural alternatives to treat obesity is a current research goal. This review therefore examined the anti-obesity potential of natural chalcones based on available evidence from in vitro and animal studies. In particular, the results of the main in vitro studies describing the principal molecular therapeutic targets and the mechanism of action of the different chalcones investigated were described. In addition, the results of the most relevant animal studies were reported. Undoubtedly, future clinical studies are urgently needed to confirm and validate the potential of natural chalcones in the clinical prophylaxis of obesity.

## 1. Introduction

In recent years, the growing prevalence of obesity has become a major global health concern, demanding urgent attention and effective strategies to contrast this multifaceted pandemic issue [1]. Clinically, obesity is defined as a persistent state of positive energy balance between energy intake and expenditure, resulting in an excessive accumulation of fat mass, especially visceral fat, which leads to an increase in the body mass index (BMI) value. As reported by the World Health Organization (WHO), BMI values between 25 and 29.9 are indicative of overweight or pre-obese conditions, while BMI ≥ 30 is predictive of conclamant obesity [1,2]. Therefore, the occurrence of this chronic disorder was not only linked to dietary alterations, but also to the combination of several other parameters including environmental factors, Western lifestyle, and hereditary factors. Additionally, obesity has several serious public health implications mainly related to the fact that this pathology is positively correlated to the onset and progression of several chronic diseases, including type 2 diabetes (T2D), hypertension, cardiovascular disease, congestive heart failure, stroke, rheumatoid arthritis, insulin resistance, and cancer [3,4]. In this regard, it was estimated that 44% of cases of type 2 diabetes, 23% of cases of ischemic heart episodes, and up to 41% of diagnosticated cancers are attributable to the obesity/overweight condition [5]. Moreover, obesity could also lead to psychological problems, such as depression and low self-esteem [1].

Traditionally, the first multi-disciplinary approaches for obesity treatment were based on diet modification and regular exercise, but when this intervention is not enough to contrast serious obesity conditions, the treatment may rely on several pharmacological remedies [6]. The conventional drug approach to obesity management is based on different classes of molecules, including appetite suppressants, lipase inhibitors, hormones, metabolic regulators, and inhibitors of gut peptide receptors. Each one of these molecule classes is characterized by a completely different mechanism of action, reflecting the multifactorial etiology of obesity diseases. For example, phentermine, diethylpropion, and liraglutide, a sympathomimetic amine, are synthetic drugs that act at the central nervous system (CNS) level, where they regulate appetite and satiety. Orlistat is a well-known inhibitor of pancreatic lipase [7], used primarily for weight control since its activity reduces the absorption of dietary fat at the intestinal level. Hormonal regulators, instead, are a class of anti-obesity drugs that target hormonal pathways involved in appetite regulation and energy balance. Examples include the GLP-1 (glucagon-like peptide) receptor agonists such as liraglutide and semaglutide, which increase satiety and promote weight loss. Finally, the last class is metabolic modulators, which include molecules able to alter metabolic processes to promote weight loss. Unfortunately, all the above-considered pharmacological treatments are associated with serious side effects. In this regard, phentermine is available only for short-term treatment in most countries, because prolonged usage could cause a serious case of tolerance and dependency [8]. In addition, orlistat treatment is associated with various gastrointestinal side effects including oily stools, oily spotting, and flatulence [9]. In light of these considerations, researchers are increasingly turning to the world of natural compounds to seek new and safe possibilities to contrast this complex disorder, in line with what has already been carried out for the treatment of other pathological conditions with major social implications, such as hypercholesterolemia [10], diabetes [11,12,13], cardiovascular diseases [14,15], oxidative stress [16,17], and inflammation [18,19,20].

Recently, among natural promising compounds, chalcones, a class of natural flavonoids, attracted scientific interest due to their anti-inflammatory [21], anticancer [22], and antimicrobial effects [23]. Specifically, in nutraceutical research, this class of molecules has attracted considerable attention, especially to its remarkable antidiabetic activity, where phloretin and phlorizin are the main representatives [24,25].

**Figure 1:**
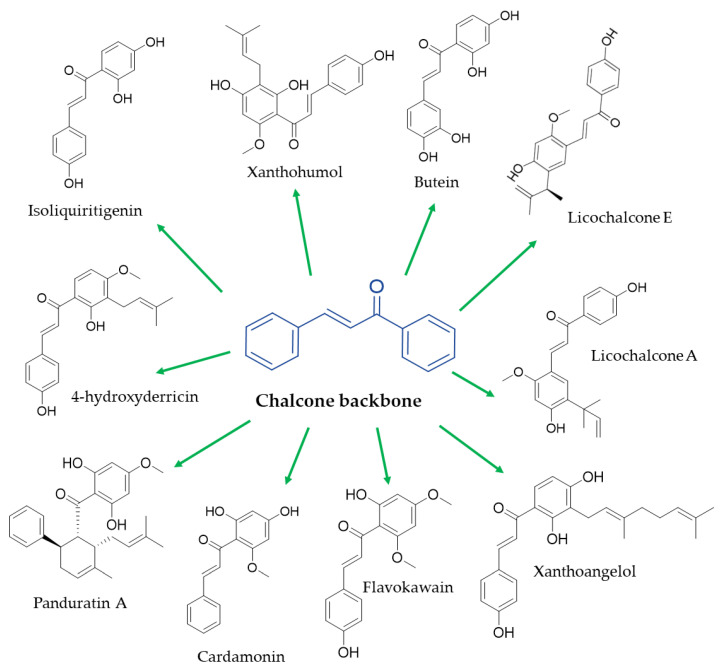
Chemical structure of the main natural chalcones with anti-obesity potential.

Chemically, chalcones are derivatives of aromatic ketones, 1,3-diphenyl-2-propen-1-ones, a precursor of flavonoid biosynthesis [26]. Based on the type of subtraction, chalcones are generally classified into hydroxy chalcones, methoxy chalcones, amino chalcones, aryl chalcones, alkyl chalcones, nitrogenous chalcones, and other subclasses less representative [22] (Figure 1). Considering that the simple chalcone structural scaffold can be functionalized at multiple sites with different chemical moieties, these molecules could respond to different molecular targets or interfere with different signaling pathways in humans with a specific structure–activity relationship [27].

Recently, this large class of molecules has demonstrated interesting and promising anti-obesity effects in both in vitro and in vivo systems. Thus, the aim of the present study was to synthesize the available scientific knowledge about the natural chalcone anti-obesity potential, shedding light on their molecular mechanisms through the analysis of in vitro and in vivo studies conducted thus far.

## 2. In Vitro Evidence

### 2.1. Cardamonin

Cardamonin ((2E)-1-(2,4-dihydroxy-6-methoxyphenyl)-3-phenyl-2-propen-1-one) is a dietary polyphenol, belonging to the class of chalcones and mainly isolated from *Alpinia* species [28]. A wide range of biological activities are referred to this molecule, including anti-inflammatory and antitumor activity [28,29]. Recently, this compound has attracted considerable attention due to its potential anti-obesity potential. Specifically, in a recent comparative study, 46 different polyphenols were tested on the mouse embryonic fibroblast 3T3-L1 cell model (well-established models for studying the differentiation of preadipocytes to mature adipocytes, i.e., adipogenesis [30]) in order to evaluate their effects on intracellular lipid accumulation. Specifically, among all the tested polyphenols, the authors described that cardamonin was one of the most active in contrasting the differentiation of preadipocytes in mature adipocytes by the activation of a specific molecular pathway [31]. Specifically, at a molecular level, the preadipocyte maturation process could be divided into four consecutive steps: growth arrest, clonal expansion, early differentiation, and final differentiation. These phases are modulated with a complex framework of transcription factors, which coordinates the production of several proteins responsible for establishing the mature adipocyte phenotype, involving peroxisome proliferator-activated receptor (PPAR)γ, fatty-acid-binding protein 4 (FABP4), and CCAAT/enhancer-binding proteins (C/EBP) α. During adipogenesis, the initial expression of C/EBPβ promotes the synthesis of C/EBPα and PPARγ, and these two proteins work together to maintain the differentiated phenotype [32]. In addition to these intracellular pro-obesity master regulators, the obesity progression could be controlled by the regulation of an extracellular signal-regulated kinase (ERK) expression, a member of the mitogen-activated protein kinase family, which modulates several important physiological processes at the cellular level. Particularly, in preadipocytes, the activation of ERK is an essential stage in adipocyte differentiation, and its activation induces the phosphorylation of PPARγ, leading to attenuation of the differentiation process. In this regard, the authors described that cardamonin was not only able to inhibit adipocyte differentiation by activating the ERK pathway, leading to a downregulation of all adipocyte transcription factors, but also to reduce the specific adipokine production normally released by the mature adipose tissue [31]. Adipokines are multitasked molecules deeply implicated in lipid metabolism, the adipogenesis process, inflammation (reactive protein C), and regulation of energy balance [33]. Furthermore, other authors have reported that cardamonin could also interfere with other molecular pathways implicated in obesity progression. According to a recent study conducted by Young-Jin et al., cardamonin treatment could inhibit lipogenesis by activating protein-kinase-A-mediated browning in the 3T3-L1 cell line. In mammals, the energy equilibrium is regulated by the presence and secretion of two different types of adipose tissue: white adipose tissue (WAT) and brown adipose tissue (BAT) [34]. Physiologically, the energy surplus is stored as triglycerides in WAT, which is composed of adipocytes containing large unilocular lipid droplets [35]. On the other side, BAT is instead formed by adipocytes containing numerous small multilocular lipid droplets, demonstrating high levels of mitochondrial biogenesis that dissipates energy as heat due to it expressing uncoupling protein 1 (UCP1) in the internal mitochondrial membranes. Recently, in view of finding remedies for obesity prevention and treatment, great attention has focused on the browning process, which converts white adipocytes to brown-like (beige or brite) adipocytes [36]. The beige adipocytes, as well as the brown adipocytes, are characterized by high mitochondrial activity [37], which leads to increased energy expenditure that may prevent obesity onset and progression. The WAT browning process at the molecular level is modulated by the expression of several brown-adipocyte-specific factors such as PR domain containing 16 (PRDM16), peroxisome proliferator-activated receptor gamma coactivator 1-alpha (PGC1α), and UCP1. In light of these considerations, the authors described that cardamonin induced the expression of the browning marker genes PRDM16, PGC1α, and UCP1 at the mRNA and protein levels. The activation of protein kinase A (PKA) causes the activation of lipolytic enzymes, such as adipose triglyceride lipase (ATGL) and hormone-sensitive lipase (HSL), inducing lipolysis and inhibiting lipid accumulation. Generally, the term lipolysis refers to the hydrolysis of triacylglycerol (TG), which releases free fatty acids (FFAs) that could undergo mitochondrial β-oxidation following trans-membrane transport by carnitine palmitoyltransferase 1 (CPT1). The oxidation of FFA resulted in acetyl-coA, which in turn inhibited the conversion of FFA in TG, but released FFA was used to produce ATP, and consequently led to a reduction in lipid accumulation. Thus, the PKA could be a valuable regulation checkpoint for the browning process. In addition, Seo and collaborators confirmed the cardamonin inhibitory activity on the expression of the adipogenic proteins C/EBPα and FABP4 in a preadipocyte cell line pretreated with differentiation inducers. Furthermore, they have also investigated the cardamomin potential in downregulating the expression of other lipogenic proteins, such as lysophosphatidic acid acyltransferase theta (LPAATθ), lipin 1, diacylglycerol acyltransferase 1 (DGAT1), sterol regulatory element-binding protein 1 (SREBP1), and fatty acid synthase (FAS). Each one of these proteins is differently implicated in lipidic metabolism in the adipocyte; specifically, LPAATθ and DGAT1 are directly involved in triacylglycerol synthesis, while SREBP1 and FAS modulate fatty acid synthesis [38].

### 2.2. Licochalcone A

Licochalcone A (LA) (4′,4-Dihydroxy-3-α,α-dimethylallyl-6-methoxychalcone) is the primary active compound of *Glycyrrhiza inflata* and *Brassica rapa* L. Although LA has been shown to have multiple human pharmacological effects such as anti-inflammatory, antitumor, antifungal, and antiparasitic properties [39], its anti-obesity potentiality was poorly reported. Quan et al. have described that LA treatment on 3T3-L1 preadipocytes reduced lipid accumulation and downregulated the expression of several factors implicated in adipocyte differentiation and lipogenesis, such as PPARγ and C/EBPα, and in addition, limited the expression of SREBP1c. The reduction in the expression rate of these mediators regulates the fatty acid synthesis (fatty-acid-binding protein, FAS, stearoyl-CoA desaturase 1 (SCD1), and glycerol-3-phosphate acyltransferase (GPAT)) [40]. Moreover, the authors have highlighted LA positive activity also at the mitochondrial level, since the LA treatment on the 3T3-L1 preadipocyte stimulates the gene expression of CPT1, the trans-membrane mitochondrial proteins, responsible for the FFA translocation at the mitochondrial level, reasonably because of collateral inhibition of acetyl-CoA carboxylase (ACC), which is involved in the endogenous fatty synthesis, via AMP-activated protein kinase (AMPK) or the activation of nuclear receptor PPAR-α [40]. Other authors have also investigated the capacity of LA to induce the browning process in the 3T3-L1 cell line. Specifically, they found that LA treatment was able to upregulate the expression of UCP1, an important browning modulator in 3T3-L1 adipocytes [41]. Other authors have investigated the LA in vitro inhibition activity on the pancreatic lipase enzyme. Specifically, in a study conducted by Won and colleagues, LA showed relevant lipase inhibitory activity, with calculated IC_50_ values of 35 mg/mL (103.4 mM). Particularly, LA-pancreatic lipase inhibition was shown to be a non-competitive inhibition, and there was an estimated K_i_ of 11.2 mg/mL (32.8 mM), with unvaried k_m_ values for overall different concentrations tested [42]. Other authors have conducted a comparative study on the ability of different natural lichochalcones, licochalcone A, licochalcone D, and licochalcone E, to inhibit pancreatic lipase activity. Structurally, these molecules differ in the subtraction type in C2 at the level of the A ring, with licochalone D containing an isopropyl group, licochalone E containing a propyl group and no isopentyl group on the B ring, whereas licochalone A contains a hydroxy group [43]. All these molecules have shown a relevant inhibition of pancreatic lipase activity, resulting in a calculated IC_50_ of 4.9, 3.2, and 5.8 μM, respectively [44]. In addition, the performed structure–activity relationship study also indicated that the presence of both isopentenyl and hydroxyl groups at the A ring level was essential for the noncovalent inhibitory potency of the natural chalcones investigated [44].

### 2.3. Butein

Butein (3,4,2′,4′-tetrahydroxychalcone) is a natural chalcone primarily isolated from *Toxicodendron vernicifluum,* a traditional Chinese lacquer tree [45]. Yang et al. revealed that butein treatment inhibited lipid accumulation and adipogenesis in the 3T3-L1 preadipocyte cell line, by downregulating the adipogenic factors PPARγ and C/EBP-α [46]. The authors described that butein at concentrations of 5, 10, and 25 μM decreased PPARγ expression by 78.8, 68.3, and 31.4%, and C/EBPα concentration by 87.3, 71.7, and 42.1% in 3T3-L1 preadipocytes’ cell line [46]. The authors described that these results are related to butein capacity to positively modulate the expression of nuclear transcription factor erythroid 2-related factor 2 (Nrf2), a crucial transcription factor, involved in the regulation of several cellular pathways. Mainly, Nrf2 directly upregulates the key C/EBP-α and PPARγ, which as previously described, are involved in maintaining the differentiated phenotype of preadipocytes [47,48]; on the other side, Nrf2 is a fundamental transcription factor playing a crucial role in maintaining the homeostatic redox tissue condition. Particularly, Nrf2 modulates the production of many enzymes involved in oxidative stress, such as thioredoxin, glutathione, heme oxygenase-1 (HO-1), quinone oxidoreductase (NQO1) as an antioxidative enzyme, and glutathione S transferases (GSTs) as detoxifying enzymes [49]. Generally, HO-1 is well known to play a key role in antioxidant processes. This enzyme catalyzes the rate-limiting stage of free heme degradation into iron, carbon monoxide, and biliverdin, all metabolites with powerful antioxidant activity [50]. Recently, several studies highlighted the HO-1 critical role in the management of metabolic homeostasis, considering its high expression level in the white adipose tissue of genetic or high-fat diet (HFD)-induced obese mice [51]. Therefore, Nrf2 and its downstream enzyme HO-1 may be a potential target in treating obesity-related diseases. In this regard, Yang et al. have demonstrated that butein treatment on 3T3-L1 results in increased expression of Nrf2, which in turn leads to upregulation of the HO-1 mRNA expression level. A similar experimental design was followed by Wang et al., who also confirmed butein capacity to induce the upregulated HO-1 mRNA expression and the related protein expression in the 3T3-L1 adipocyte cell line [52]. Additionally, Song et al. conducted a comparative study on the anti-obesity potential of different natural molecules isolated from *Rhus verniciflua* Stokes, a lacquer tree with ancient traditional medical usage [53]. They reported that preincubated C3H10T1/2 cells with adipogenic inducer mixtures were treated with 10 mM of each different molecule studied, in order to investigate their anti-adipogenic activity. Interestingly, they found that butein suppressed lipid umulation and morphological changes during adipocyte differentiation through the suppression in a dose-dependent manner of PPARγ, over the butein concentrations of 10–40 mM. To confirm the butein antiangiogenic potentiality, the authors also compared the butein activity to those obtained upon the treatment with two well-known and established anti-obesity natural molecules, i.e., resveratrol and genistein. Interestingly, they reported that the inhibition of lipid accumulation in C3H10T1/2 cells with resveratrol treatment was obtained at the concentration of 20 µM, with genistein at 40 µM, and surprisingly with butein at 10 µM [53]. Moreover, the mRNA expression level of the main adipocyte markers, such as PPARγ, aP2, and LPL, upon the pretreatment with resveratrol, genistein, and butein was also investigated. The results indicated that butein was able to reduce the expression of 50% of these obesity markers at the concentration of only 5 µM, while the same results were achieved with 20 and 40 µM of resveratrol and genistein, respectively. In addition, butein at the dose of 10 µM completely suppresses the expression of the above-mentioned agents [53]. To better understand the molecular mechanism responsible for butein anti-adipogenic effects, the same authors have studied on the 3T3-L1 cell model the capacity of butein to modulate STAT-3, a factor highly required for adipocyte differentiation. Specifically, they reported that after the treatment for 12 h and 24 h of 3T3-L1 with butein, the expression of the main STAT3-regulated genes (*KLF5* and *P53*) was downregulated. Moreover, other authors have studied the butein mechanisms of inhibition of the obesity process by regulating AMPK, a serine/threonine protein kinase, involved in a molecular pathway that is generally recognized as an important target for obesity management [54]. Physiologically, AMPK acts directly in the modulation of cellular energy balance during the 3T3-L1 cell differentiation, by regulating lipid and glucose metabolism [55]. More specifically, the activation of AMPK leads to the attenuation of expression of the previously cited transcription factors (C/EBPα and PPARγ), but also inhibits the expression of GPAT-1 and increases the expression of CPT-1. This enzyme is involved in the endogenous synthesis of fatty acid, and at the same time inhibits the oxidation of fatty acid through the inactivation of ACC, as previously described being involved in endogenous lipid synthesis [41]. In this regard, Lim et al. demonstrated that butein treatment of 3T3-L1 cells, in addition to the effects on the C/EBPα and PPARγ expression level also described by others, was able to induce the phosphorylation of AMPK and enhances the level of phosphorylated ACC, a downstream substrate of AMPK, in a dose-dependent manner [56].

### 2.4. Panduratin A

Panduratin A (PAN A) is a prenylated flavonoid based on a chalcone skeleton with three oxygenated patterns only at the level of the B ring and a geranyl substituent at the C2-C3 position with the Diels–Alder reaction [57]. This natural chalcone was mainly isolated from *Boesenbergia pandurata* (Roxb.) Schltr. (Syn. *Kaempferia pandurata* Roxb.)*,* an ancient medicinal plant, largely used for its relevant anti-inflammatory and antioxidant properties. In this context, Kim and colleagues [58] have studied the potentiality of PAN A as a novel AMPK activator. As previously described in Section 2.3, the regulation of AMPK could be a useful tool for the treatment of obesity due to its important role as an energy sensor at the mammalian cellular level. Thus, they reported that the PAN A treatment on different cellular models used (3T3-L1 adipocytes, HepG2 liver carcinoma cells, and L6 skeletal muscle cells) induced the AMPK-dependent inactivation of ACC, resulting in a decrease in endogenous fat synthesis. More specifically, AMPK could be activated by several modulators including LKB1, CaMKKβ, sirtuin (SIRT 1), and NAD(P)H. Therefore, in order to elucidate the molecular mechanism of AMPK activation by PAN A, the authors investigated the effects of this natural chalcone on the expression of AMPK activators. The authors found that PAN A treatment increased LKB1 translocation from the nucleus to the cytoplasm and the binding rate to AMPK [58], resulting in its rapid activation. Thus, they assert that the AMPK activation measured was an LKB1-dependent process. Beyond the effects on AMPK induction, PAN A treatment on one side induces a remarkable downregulation of pro-adipogenic mediators (PPARγ, C/EBPα, ACC, FAS, and SREBP1c); on the other side, it induces an upregulation of the expression of genes involved in fatty acid oxidation (PPARα, PGC-1α, CPT-1L, CPT-1M, and UCPs) in all the cell models used (3T3-L1, HepG2, and L6 cells). These results are in line with the findings of other authors, who have observed that PAN A treatment of 3T3-L1 and HepG2 cell lines decreased triglyceride accumulation via activating the AMPK pathway [59].

### 2.5. Isoliquiritigenin

Isoliquiritigenin (ILG, 2′,4′,4′-trihydroxy chalcone), a chalcone compound belonging to the flavonoid family, consists of two benzene rings linked through an α,β-unsaturated carbonyl group [60]. ILG is usually isolated from *Glycyrrhiza uralensis* (licorice), *Allium ascalonicum*, *Astragalus membranaceus*, *Dalbergia odorifera*, *Dianthuschinensis*, *Glycine max* L., *Sinofranchetia chinensis* [60,61,62]. This molecule was largely studied for its relevant health-promoting effects, including anticancer, anti-inflammatory, antimicrobial, antioxidative, antiviral, antidiabetic, and anti-depressive activity [63]. In a study conducted by Zeng et al. (2022), it was reported that ILG is advantageously associated with diminishing cholesterol levels in different cellular models [64]. The mechanism proved to be assertive through downregulation of expression of Niemann Pick C1 Like 1 (NPC1L1), which is a protein responsible for intestinal absorption of cholesterol. Interestingly, NPC1L1 is not only a relevant molecular target for hyperlipidemia therapy but also for obesity management, considering that the cholesterol uptake rate is known to be one of the main elevating factors of obesity. In this regard, Zeng and colleagues [64] used the colon cancer cell line (Caco-2) and liver cancer cell line (HepG2) cell model to explore the ILG-NPC1L1 downregulatory activity. Auspiciously, they observed that this natural product (at the dose of 100 μmol/L) was as efficient as the pharmaceutical complex of Ezetimibe (EZ, used as positive control) in the limitation of the cholesterol uptake rate. It is known that EZ is marketed as a cholesterol absorption inhibitor that takes over the function of NPC1L1 and also has some pleiotropic effects [65]. Furthermore, the authors reported that in both the cellular models, NPC1L1 expression was modulated by ILG treatment in a dose-dependent manner, and at the same treatment dose, the higher inhibitory effects were observed in Caco-2 cells in comparison with the HepG2 cells. Additionally, the anti-obesity function of ILG was explored in another study accomplished by Park et al. (2016) [66]. Specifically, they explored the capacity of ILG, at two different dosages (75 or 100 µM), to regulate the insulin-stimulated adipogenesis in 3T3-L1 cells. It is known that adipocyte maturation is strongly regulated by the insulin signaling pathway [67]. Insulin is one of the most important adipogenic hormones responsible for modulating several transcription factors involved in adipocyte differentiation and lipid accumulation, such as PPARγ C/EBPα, as well as the expression of profibrogenic genes, such as FABP4, also known as aP2, and the translocation of glucose transporter 4 (GLUT4) at the adipocyte level. The authors described that not only was ILG able to inhibit the production of all the above-mentioned adipogenic molecular agents, but was especially able to inhibit the insulin-stimulated adipocyte differentiation through the regulation of oxygen species (ROS) production that occurs during this process. Specifically, increased production of intracellular ROS occurs during the initial phase of insulin-stimulated adipogenesis, leading to the activation of NADPH oxidase 4. ROS blocked the activation of protein-tyrosine phosphatase 1B (PTP1B), which is a pivotal modulator of phosphorylation-dependent insulin signaling, resulting in an increased adipocyte differentiation rate. The authors demonstrated that ILG in virtue of its intrinsic antioxidant potential (related to two functional hydroxyl groups) was able to inhibit the oxidation of PTP1B, and consequently the activation of its related downstream signaling pathway [68]. In addition, Moon et al. (2020) [69] evaluated the impact of low-dose ILG treatment on the differentiation process of white adipocytes into functional beige ones in human adipose-derived stem cells (hASCs), stem cell lines directly isolated from human fat tissue. The following was established well: the crucial roles of brown adipocytes (mitochondria-rich multilocular cells) and beige adipocytes of subcutaneous white adipose tissue containing thermogenin (UCP1) in energy dissipation, lipid and glucose oxidation, and uncoupling the mitochondrial respiration [70]. The authors reported that ILG treatment promotes brown adipogenesis by increasing the expression of both UCP1 and PRDM16, as previously described with two relevant browning markers. In addition, the authors reported that the ILG treatment led to a downregulation of the c-Jun N-terminal kinase (JNK) expression rate, a specific kinase implicated in adipocyte differentiation. Finally, the authors assessed that low-dose ILG induced the beige adipocyte potential of hASCs via JNK inhibition. Additionally, the inhibition of monoamine oxygenase-B (MAO-B), a key enzyme for cholinergic transmission, could find a therapeutic application for obesity management [71]. Specifically, MAO-B inhibitors have, to an unheard-of degree, shown a relevant anti-obesity potential via the reduction in adipose tissue, and promotion of weight loss. In light of these considerations, Prajapati and colleagues investigated the ILG capacity to inhibit the enzymatic activity of human monoamine oxidase (hMAO-A and hMAO-B) in a vitro enzymatic assay [72]. The authors reported that ILG was able to contrast the MAO-A and MAO-B enzymatic activity with calculated IC_50_ values of 0.68 and 0.33 µM, respectively. Additionally, the conducted study on the enzymatic kinetic mechanism indicated that ILG was able to inhibit the MAO activity in a competitive way.

### 2.6. Xanthohumol

Xanthohumol (XN, 3′-(3,3-dimethylallyl)-2′,4′,4-trihydroxy-6′-methoxychalcone) is a natural prenylated chalcone, mainly extracted from the hops plant, *Humulus lupus*. Samuels and colleagues investigated the XN anti-obesity potential on 3T3-L1 adipocytes and primary human subcutaneous preadipocyte models [73]. According to their experimental protocol, initially, in both the cell systems used, the differentiation was induced, followed by treatment with XN at different concentration levels, dorsomorphin as an AMPK inhibitor (positive control), or 5-Amionimidazole-4-carboxamide (AICAR) as an AMPK activator. Their results indicate that XN acts as a bridging agent in white adipocytes as suggested with the increased synthesis of beige markers CIDE-A and TBX-1. After 24 h of treatment, XN activated AMPK, leading to the upregulation of UCP1, phosphorylation of ACC, and HSL expression. In addition, they reported that XN acts positively at the mitochondrial level, inducing mitochondrial biogenesis, as evidenced with increased mitochondrial content, enhanced expression of PGC-1α, and the thermogenic protein UCP1 [73]. In addition, Miyata et al. investigated the ability of XN in Huh7 cells, a human hepatoma line, to inhibit SREBP, by impairing the translocation of the SREBP cleavage-activating protein complex from the endoplasmic reticulum to Golgi [74]. Instead, Rayalam and colleagues have studied the XN obesity potential in combination with another natural compound, guggulsterone (GS), in 3T3-L1 adipocytes [75]. They reported that XN and GS, tested separately at a treatment dose of 1.5 mM (XN) and 3.12 mM (GS), can each reduce lipid accumulation by 26 ± 4.5% (*p* < 0.001), while their combination results in a 78.2 ± 1.8% (*p* < 0.001) reduction in lipid accumulation, indicating possible synergistic effects. Additionally, a similar XB effect on 3T3-L1 adipocytes was also reported by Rossi et al. [76], who described that XN up to 50 µM could reduce adipocyte proliferation, but that this resulted in adipocyte hypertrophy without an improvement in the metabolic profile.

### 2.7. Others

Flavokawains A, B, and C are a member of the natural chalcones family mainly isolated from *Kaempferia angustifolia*, a plant belonging to the ginger species. Hanif and colleagues have studied the capacity of pure flavokawains A, B, and C to inhibit the differentiation of murine 3T3-L1 preadipocytes [77]. Their results indicate that flavokawains A, B, and C modulate the adipocyte differentiation with a calculated IC_50_ (µM) of 74.8, 23.1, and 41.8, respectively. Moreover, Zhang et al. demonstrated the anti-obesity potential of flavokawains B in combination with another natural chalcone, cardamonin [31]. As previously reported for cardamonin, flavokawains B also increased the phosphorylation of ERK and inhibited lipid accumulation by downregulating the expression of CCAAT/enhancer-binding protein (C/EBP)-β, C/EBPα, and PPARγ at both mRNA and protein levels [31]. Interestingly, another study investigated the anti-obesity potential of two natural chalcones, Xanthoangelol and 4-Hydroxyderricin. These two compounds are isolated from *Angelica keiskei* (Japanese name: “Ashitaba”), a Japanese herb, largely used in traditional medicine, with several purposes, and anti-inflammatory, antitumor, and antidiabetic activity. Zhang and his research group have investigated the effects of the combination of these two molecules on adipocyte differentiation in the 3T3-L1 cell line. They reported that these compounds (1, 5, 10, and 30 µM) inhibited adipocyte differentiation, inducing a down-expression of the above-cited adipocyte-specific transcription factors, C/EBP and PPARγ. These two molecules induced the phosphorylation of AMPK and acetyl CoA carboxylase during differentiation of 3T3-L1 adipocytes coupled with a reduction in glycerol-3-phosphate acyl transferase-1 and an increase in carnitine palmitoyltransferase-1 mRNA expression [78]. Additionally, the MAO inhibitory activity of Xanthoangelol and 4-Hydroxyderricin, isolated from the same plant matrix, was further investigated by other researchers. They described that Xanthoangelol is a nonselective MAO inhibitor, with an estimated IC_50_ value to MAO-A and MAO-B of 43.4 μM and 43.9 μM, respectively. In contrast to 4-Hydroxyderricin, it shows a potent capacity to selectively inhibit MAO-B. The calculated IC_50_ value of 4-Hydroxyderricin to MAO-B was 3.43 μM, which is higher than that of deprenyl (0.046 μM) used as a positive control for selective MAO-B inhibition [79].

## 3. In Vivo Studies

### 3.1. Cardamonin Derivatives

The DMC (2′,4′-dihydroxy-6′-methoxy-3′,5′-dimethylchalcone), a cardamonin close structural analog, was studied by Choi and colleagues to investigate its anti-obesity potential. They performed an in vivo study using a high-fat diet (HFD)-induced obesity mice model [80]. Specifically, C57BL/6 HFD male mice were treated with or without (control) 30 mg/kg/day of DMC, administered using oral gavage for 3 weeks. No change in body weight was observed in the DMC group in comparison to the control group, but, when comparing white adipose tissue (epididymis, retroperitoneal, subcutaneous) weight to the total body weight, a lower ratio was observed in DMC-treated mice. Additionally, the intra-peritoneal glucose tolerance test showed a higher glucose tolerance in the DMC-treated group. Furthermore, DMC administration led to a marked increase in fatty acid oxidation mediated with an increased level of AMPK phosphorylation in the muscle tissue of DMC-treated mice in comparison to the control group. Finally, the authors asserted that the DMC effect on adipogenesis enhanced obesity and glucose tolerance in the in vivo HFD-mice model through the induction of fatty acid oxidation, which is mediated with AMPK activation in muscle. The DMC capacity to modulate in vivo this important molecular pathway was further investigated by others. In 2018, Hsieh et al. reported similar effects with two structurally close synthetic halogen-containing chalcone derivatives: 2-bromo-4′-methoxychalcone and 2-iodo-4′-methoxychalcone. To assess the anti-obesity potential, HFD C57BL/6J mice were administered these two synthetic chalcones [81]. After the treatment period (10 weeks), these compounds revealed the ability to inhibit body weight gain and to induce a reduction in the adipocyte cell size in HFD mice via AMPK activation.

### 3.2. Butein

To investigate butein anti-obesity potential, Wang et al. (2017) demonstrated butein induction of heme oxygenase (HO)-1 expression in adipocytes in in vitro and in vivo models. This enzyme has an important role in metabolic homeostasis since its presence in adipocytes protects against obesity and adipose tissue dysfunction, as previously described in Section 2.3. The study was conducted on 6-week-old male C57BL/6 mice fed a normal diet (control group) or a 60% high-fat diet (HFD). Butein (10 mg/kg) or HO-1 inhibitor SnPP (tin protoporphyrin IX) (10 mg/kg) was administered intraperitoneally to HFD mice three times a week for 3 weeks. Butein-HFD mice showed lower body weight and decreased blood glucose levels as compared to the HFD group. Butein administration also induced a marked decrease in epididymal fat mass, with smaller adipocyte cell size. In addition, in order to investigate which are the molecular mediators responsible for favorable observed results, the authors have also studied the gene expression in mice eWAT (epididymal white adipose tissue). The results indicate that, in butein-treated eWAT, an upregulation of HO-1 and NQO-1 protein expression was observed, consistent with the in vitro effect previously described. Moreover, it was found that butein treatment represses inflammatory genes (Il-6, TNF-α, and Mcp-1) in eWAT via HO-1 induction. These results assessed butein potential for repressing obesity-induced glucose intolerance, adipose hypertrophy, and inflammation, and reducing adipose tissue mass in HFD mice, through HO-1 activation in adipose tissue. Interestingly, in another study, butein treatment was able to induce the browning of WAT, through the induction of two master browning relators, PRDM4 and UCP1, in a mice model [82]. Specifically, the study was conducted on three groups of mice (*n* = 7 per each group), involving control, low-dose treatment (5 mg/kg), and high-dose treatment (15 mg/kg), for about 2 months. After the treatment period, butein enhanced the expression of PRDM4 and UCP1 in inguinal white adipose tissues (iWATs), epididymal white adipose tissues (eWATs), and brown adipose tissues (BATs). PRDM4 and UCP1 induce the development of beige adipocytes in WAT, inducing thermogenesis, and energy dissipation. On the contrary, Hemmeryckx et al. reported an in vivo study that showed that butein administration did not change UCP1 expression and did not induce adipose tissue browning, probably due to a too-low dose tested and too-short time exposure [83]. Particularly, they conducted two types of in vivo studies, the first one performed on male hemizygous mice for Tg(Ucp1-luc2,-tdTomato)1Kajim(ThermoMouse) treated with a vehicle (control) or butein at 10 or 20 mg/kg for 4 days, injected intraperitonally, in order to investigate the butein effects on UCP1 expression using a luciferase-based assay. The second type of experiment was conducted on C57BL6/Rj mice fed a methionine- and choline-deficient diet (MCD), supplied intraperitonally with a vehicle or butein at 20 mg/kg, for a single month. In the ThermoMouse model, butein administration showed no effect on UCP1 expression in adipose tissue, total body weight, subcutaneous inguinal and intra-abdominal gonadal (GON) fat, and interscapular brown adipose tissue (BAT). Similar results were obtained with the administration of butein in MCD-fed C57BL6/J mice, which also showed no change in liver triglyceride content. In conclusion, butein administration did not improve UCP1 expression and did induce browning of adipose tissue, which may be because a longer treatment and/or higher doses of butein are needed. The potential butein anti-obesity activity was also studied by Farias-Pereira et al., who evaluated the butein capacity to modulate lipid metabolism in adult nematodes (*Caenorhabditis elegans*) [84]. According to their experimental protocols, adult nematodes were treated for 2 days with 35 or 70 μM of butein. The obtained results indicate that butein at 70 μM decreased triglyceride content by 27% compared to the control, without changing food intake and energy expenditure. In order to investigate the triglyceride reduction molecular mechanism, the authors studied the effects of butein on lipid-metabolism-related transcription factors and enzymes, and via the SKN-1 pathway. The results suggest that butein acts as a lipogenesis inhibitor via SKN-1- and FAT-7-dependent pathways in *C. elegans*. Specifically, butein downregulates sbp-1 (SREBPs’ homolog in *C. elegans*) and fat-7 (the homolog of stearoyl-CoA desaturase, an acid-Δ9-desaturase with anti-obesity activity in *C. elegans*). In conclusion, butein decreased triglyceride content by inhibiting lipogenesis in *C. elegans*.

### 3.3. Licochalcone A

The anti-obesity potential of licochalcone A (LA) was evaluated in a study reported by Lee et al. (2018). They reported an effect of *Glycyrrhiza uralensis* and its ingredients in the modulation of adipogenesis through adipocyte browning using a high-fat diet (HFD)-induced obesity mice model. The browning process consists in the development of brown adipocyte tissue (BAT) within white adipose tissue (WAT) in response to endogenous or exogenous stimuli. BAT is rich in mitochondria with expression of UCP1, an important thermogenin that stimulates heat generation. According to the experimental model used, C57BL/6 mice were fed HFD (40% kcal) and treated with 10 mg/kg of LA, intraperitoneally administered, once per day for 19 days. At the end of the treatment, LA induced a marked decrease in body weight gain (from 38.25 ± 0.79 g to 31.31 ± 1.14 g) and inguinal adipose tissue weights. In addition, lower serum levels of glucose and total cholesterol were observed in HFD mice administered LA, proving that LA ameliorated metabolic disorders induced with HFD. In order to understand the mechanism that led to body weight reduction, the UCP1 expression level was investigated in inguinal white adipose tissue (iWAT) isolated from treated mice. LA administration increased the expression of browning-specific markers, such as UCP1, PRDM16, and PPARγ coactivator-1 (PGC-1α) in iWAT. Moreover, LA induced a shift of the white adipocyte phenotype to brown adipocyte phenotype in iWAT, since HFD-LicoA iWAT was characterized by multilocular adipocytes with UCP1 expression, while HFD mice iWAT was characterized by unilocular adipocytes and low UCP1 expression levels. These results demonstrate that LA could reduce obesity and recover metabolic homeostasis by inducing the brown fat phenotype. Obesity is highly correlated with non-alcoholic fatty liver disease (NAFLD); thus, the effect of LA on hepatic lipid metabolism was evaluated on ICR mice fed with a regular diet (RD), HFD, LA 5 mg/kg HFD, and LA 10 mg/kg HFD [85]. After a treatment period of 3 weeks, a liver tissue analysis revealed that lipid and triglyceride levels in the liver tissue were significantly lower in the LA-treated groups when compared to those in the HFD control group. The authors described that the decreased triglyceride accumulation obtained could be related to AMPK activation, as AMPK and ACC were dose-dependently phosphorylated in the liver tissue with the administration of LA in HFD mice. In addition, RT-PCR and real-time PCR results, conducted on liver tissue, showed that LA supplementation decreased the transcription of SREBP1c and its target enzymes (SCD1, FAS, and GPAT) and upregulated the gene expressions of proteins such as peroxisome proliferator-activated receptor α (PPARα) and fatty acid transporter (FAT/CD36), which are responsible for lipolysis and fatty acid transport, respectively. In conclusion, it was proved that LA could be employed in the prevention and treatment of NAFLD. In line with this experimental model, Liou and colleagues [86] explored the LA capacity in reducing weight gain and improving the NAFLD condition, in a model based on male C57BL/6 mice, fed with a controlled HFD (to induce these pathological conditions). The mice were divided into three groups, control (fed with HFD), LA low-dose treatment (fed with HFD and intraperitoneally administered LA at 5 mg/kg), and high-dose treatment (fed with HFD and intraperitoneally administered LA at 10 mg/kg), for 16 weeks. After the treatment time, the authors describe a significant reduction in total body weight (−50% vs. HFD group); it also decreased the weights of inguinal and epididymal adipose tissue. Additionally, the authors described a positive effect of LA treatment of serum triglycerides, low-density lipoprotein, free fatty acids, and fasting blood glucose. In order to evaluate the LA applicability on NAFLD management, the authors have studied the effects of LA treatment on liver tissue. Normally, liver tissue is dark brown/red in non-obese mice, while it is yellowish and absent in obese mice. After treatment with LA, the color of the liver tissue changed to a yellow pigmentation, and a significant decrease in the weight of the liver tissue was observed in both treatment groups (LA at 5 mg/kg and 10 mg/kg). In addition, a specific histological analysis of the liver tissue revealed that lipid droplets and fat vacuoles were significantly reduced in the two treatment groups compared to the control. Finally, the authors reported that LA was able to suppress the expression of the transcription factors SREBP-1c, PPARγ, and FAS in liver tissue compared to the HFD group, leading to a valuable inhibition of lipogenic activity. Moreover, when the authors studied the expression of the molecular modulators implicated in the fatty acid β-oxidation pathway at the liver tissue level, they reported that administration of LA promoted CPT-1, but not CPT2 expression, compared with the HFD group, and LA treatment also stimulated CPT-1, phosphorylation of AMPKα, and the inactivation via phosphorylation of ACC-1 in comparison to the HFD group. Finally, the authors describe that LA could alleviate the expression of several lipogenic transcription factors, such as C/EBPα, Srebp-1c, and FAS.

### 3.4. Licochalcone E

In order to assess the licochalcone E (LE) effect on obesity-related diabetes, Park et al. performed an in vivo study on a C57BL/6J mice model. They fed 5-week-old male C57BL/6J mice with a 58% high-fat diet for about 2 months and selected 11 high-glucose mice making two groups of diabetic mice treated intraperitoneally for 2 weeks: the control group administrated with the vehicle and the LE group administrated with 10 mg/kg of licochalcone E. After the treatment period, a mice serum biochemical analysis showed decreased levels of blood glucose and serum triglyceride in the LE group. Furthermore, a histological analysis performed on epididymal white adipose tissue (EWAT) demonstrates a significant reduction (33.4%) of adipocyte size in the LE group. This reduction may be due to the enhanced expression of PPARγ mRNA, which is involved in glucose and lipid metabolism and in adipocyte differentiation, inducted by LE in WAT, as seen in RT-PCR analysis results. Moreover, LE treatment stimulates protein-kinase B (Akt) signaling in EWAT, which has a key role in adipogenesis. In conclusion, the improved adipocyte differentiation makes LE a therapeutical agent for the management of hyperglycemia and hyperlipidemia in a diabetic status.

### 3.5. Panduratin A

Since AMPK activation has a key role in the management of metabolic disorders, PAN A, a natural AMPK inductor (as described in the Section 2.3), was investigated for obesity treatment through an in vivo study [58]. In order to prove that PAN A could reduce obesity in mice, sixteen C57BL/6J mice underwent an HFD in order to induce obesity, and then were treated with a vehicle (control group, *n* = 8) or 50 mg/kg/day of PAN A (PAN A group, *n* = 8) with an oral zone needle, for 2 months. After the treatment period, the PAN A group revealed lower weight gain and fat pad mass (epididymal, perirenal, and subcutaneous fat) in comparison to the control group, mainly related to a smaller adipocyte size, without reducing food intake. Furthermore, serum levels of triglyceride, total cholesterol, and low-density lipoprotein cholesterol (LDL) were improved. The authors, in addition, reported that PAN A treatment also reduced fat content in the mice liver and the muscle tissue, with the activation of LKB1, AMPK, and ACC. Moreover, PAN A treatment reduced ectopic fat accumulation in the skeletal muscle of treated mice as compared to the control group, through the induction of fatty acid oxidation protein expression (PPARα, PPARγ, CPT-1M, PGC-1α, and UCP3). Additionally, mice administered with PAN A showed higher running time and distance, due to higher contents of slow-twitch myofiber markers and mitochondria in skeletal muscle. In conclusion, the authors suggested that PAN A could improve obesity conditions by increasing the muscle oxidative potential, and metabolic profiles through dual activation of AMPK and PPAR*δ*, even without exercise [87]. Similar results were obtained from Kim et al. in 2012 [59]. In a high-fat-diet-induced obesity mouse model, they administered 200 mg/kg of a *Boesenbergia pandurate* ethanolic extract (BPE), where PAN A is the main chemical component. Specifically, they found that BPE induced a decrease in body weight and serum levels of total cholesterol, LDL, and triglycerides, via AMPK activation and lipid metabolism regulation.

### 3.6. Isoliquiritigenin

The effect of treatment with ILG was evaluated in a comparative in vivo study on C57BL/6J mice fed with a normal, high-fat, and ILG-containing high-fat diet [88]. This study, which lasted for about 4 months, assessed the effectiveness of 0.02% (*w*/*w*) ILG treatment in a high-fat diet (40 kcal% fat). Supplementation of ILG led to a significant reduction in body fat mass and a valuable positive effect on lipidic plasma levels. Particularly, the authors reported an increased HDL-cholesterol/total-cholesterol ratio (HTR) in the ILG group as a result of reduced levels of non-HDL cholesterol. Moreover, ILG improved hepatic steatosis, induced with the high-fat diet, by suppressing the expression of hepatic lipogenesis genes (FAS and SCD1) and reducing the liver triglyceride and fatty acid accumulation (plasma glutamic oxaloacetic transaminase (GOT) and glutamic pyruvic transaminase (GPT)), and at the same time enhancing β-oxidation in the liver. Interestingly, the authors also reported that the blood analysis indicates a relevant decrease in terms of glucose and insulin plasma content, which leads reasonably to ameliorating insulin resistance. Additionally, ILG administration improved energy expenditure and decreased adiposity through increasing mRNA expression of thermogenic genes in interscapular brown adipose tissue (iBAT), such as UCP1, PRDM16, and NAD-dependent deacetylase sirtuin-1 (SIRT1). The overexpression of these genes induced mitochondrial biogenesis and uncoupled cellular respiration in BAT. In conclusion, a diet supplemented at 0.02% ILG in HFD mice potentially improves body fat mass, cholesterol, and NAFLD through higher energy expenditure. In order to understand how ILG could interfere with metabolic disorders, Ishibashi et al. investigated gut microbiota modification in mice [89]. In this regard, several authors reported the potential of dietary polyphenols in reducing obesity through changes in gut microbiota composition [90]. In light of these considerations, C57BL6/J mice were fed in four different ways for about 3 months: HFD (60% fat), HFD supplemented with ILG (0.5%, *w*/*w*), normal diet (ND; 10% fat), or ND supplemented with ILG (0.5%, *w*/*w*). After the treatment period, ILG-HFD mice body weights and epididymal white adipose tissue (eWAT) weights were lower as compared with the HFD group. Additionally, glucose tolerance and insulin resistance were enhanced with ILG administration. The effect of ILG on gut bacteria composition was evaluated through a specific RNA sequencing, performed on the face samples collected from the ILG-treated mice groups. The Firmicutes/Bacteoidetes ratio was lowered with ILG administration, resulting in a remodeling of gut microbiota in favor of beneficial bacteria (e.g., *Parabacteroides goldsteinii* and *Akkemansia muciniphila*, Bacteroides) rather than lactic-acid-producing species (i.e., *Lactobacillus johnsonii* and *Lactococcus lactis*, Firmicutes). Specifically, it was reported that an elevated presence of aggressive bacteria (dysbiosis) is positively correlated to obesity, while predominate populations mainly composed of *P. goldsteinii* and *A. muciniphila* are generally considered as antimetabolic disease-associated species [91,92]. In conclusion, these results were confirmed with fecal microbiome transplantation (FMT) from ILG-fed mice, proving the prevention of HFD-induced body and eWAT weight changes, glucose tolerance, and insulin resistance, and indicating the antimetabolic syndrome effects of ILG-responsive gut bacteria.

### 3.7. Xanthohumol

The potential anti-obesity activity of xanthohumol (XN) via in vivo metabolic modulation was deeply explored. It is well established that the inequality between energy intake and consumption is one of the factors leading to obesity. The key role of mitochondria in transforming macronutrients and the generation of products for transporting protons through the mitochondrial inner membrane, causing an electrochemical potential essential for ATP synthesis, is clear. Mitochondrial uncoupling (dissipation) of the proton motive force from the ATP production at ATP synthase would result in the uncontrolled catabolism of dietary macronutrients and heat formation [93]. On the other hand, the rise in the rate of basal mitochondrial uncoupling diminishes ROS generation. The uncoupling process also elevates the expression of the transcriptional coactivator named peroxisome proliferator-activated receptor-coactivator 1 (PGC-1), which plays a crucial role in mitochondrial homeostasis and the cellular adaptive stress response (ASR) to raise the ATP level. This is triggered by slight ROS formation, weakened glutathione (GSH) depletion, and electrophilic stress [94]. In this scenario, XN, which contains an electrophilic group, is able to induce a stress response and bring up the ATP production rate [95], leading to an increase in energy consumption. Considering a previous study by Legette et al. (2013) [96], in which XN treatment (16.9 mg/kg of BW being the most efficient) positively affected weight loss of high-fat-diet-fed Zucker rats and reduction in their plasma glucose level, Kirkwood and colleagues investigated the root mechanism with a metabolomics analysis and explored the fasting plasma from the same rats treated with XN and observed lower levels of products of dysfunctional lipid metabolism, specifically dicarboxylic (DC) fatty acids and acylcarnitines (ACs) [95]. Specifically, DC and ACs are usually generated in response to incomplete fatty acid oxidation in skeletal muscle; therefore, they could be considered as markers for evaluating dysfunctional lipid metabolism and fatty acid overload. In addition, they also reported a decrease in hydroperoxy (HP) fatty acid production. Additionally, the downregulatory impact of XN on SREBPs (e.g., SCD1) in the C57BL/6J mice liver was demonstrated by Miyata et al. [74]. A lower weight gain rate and minimalized weight of the liver and epididymal, mesenteric, subcutaneous, and inguinal white fat pad parallel with the lowered weight of interscapular brown fat pads were achieved in the XN-supplemented high-fat-diet-consuming group. The abdominal visceral and subcutaneous fat volumes also had undergone the effect of XN administration. The intestinal fat absorption rate was also diminished. Recently, the bioavailability and biologic effectiveness of XN were explored in an innovative study model, where XB was encapsulated in a micellar solution (Micellar Xantho-Flav-Solubilisate (EW0192/B, 10% *v*/*v*). It was well-reported that the micellar system is an effective alternative method for providing higher bioavailability of apolar molecules. In this regard, Khayyal and colleagues have studied the anti-obesity effects of an XN-micellar formulation in comparison to the XN native form, employing mice fed with a normal diet and Western-type diet (WTD; containing fatty acids, and different carbohydrates that develop metabolic disease such as obesity, diabetes, and non-alcoholic fatty liver disease (NAFLD)) [97,98]. As a result, the administered micellar solution has come out to be significantly influential in pausing weight gain and glucose intolerance. Moreover, XN exhibited various inhibitory activities toward issues associated with hepatic steatosis, e.g., downregulation of expression of chemokine monocyte chemotactic protein 1 (MCP-1) and the cytokine (CXCL1) causing (non-alcoholic) steatohepatitis (an advanced stage of fatty liver disease) and hampering hepatic infiltration progress of inflammatory cells (validated through CD3-immunohistochemical analysis). These were achieved successfully by utilizing a minimal dosage (10 times lower) compared with the necessary efficient dosage of XN in its native form.

## 4. Materials and Methods

### Search Strategy

A comprehensive literature search was conducted using the PubMed-NCBI and Google Scholar database listings for important publications. The combinations of the following keywords were used: “nutraceuticals”, “natural chalcone”, “obesity”, “antiobesity activity”, “browning”, “adipocyte differentiation”, “mechanisms of action”, lipase inhibition, and “targets of action”, “butein”, “xanthohumol”, “cardamomin”, “phloridzin”, “phloretin”, “isoliquiritin”, “isoliquiritigenin”, “pinostrobin”, “licochalcone”, “panduratin”, and “aspalathin”. A thorough screening of scientific literature databases was conducted for the preparation of the present manuscript. Specifically, we exclusively included (i) in vitro and animal-based studies of the last 10 years, and (ii) studies evaluating the effects of treatment with food-derived bioactive chalcones formulated both in nutraceuticals and food products or used as pure molecules. In addition, a detailed search for the potential anti-obesity clinical effect in humans of natural chalcones was carried out, but without a result.

## 5. Conclusions

In this review, the most relevant studies regarding the potential application of natural chalcones for obesity prevention and treatment were summarized. While the scientific literature is particularly rich in original papers, reviews, and meta-analysis studies regarding the effects of natural chalcones in the treatment of diabetes and diabetes-related diseases, less described are the effects of these molecules for the treatment of a relevant social-implicated disease, obesity. To this purpose, the most relevant papers related to in vitro evidence are summarized, underlining the possible molecular mechanism and target implicated in obesity treatment. According to the collected scientific evidence, the main natural chalcone anti-obesity mechanism was related to their capacity to inhibit adipocyte differentiation, contrast the lipase activity, decrease the intestinal cholesterol absorption with NPCL1 inhibition, and decrease the lipid accumulation at the adipocyte level (Table 1). In addition, exhaustive research about the most relevant animal-based in vivo studies was performed, underlining that the main chalcone-based anti-obesity activity was mediated with browning induction and modulation of adipokine production, which leads to a significant weight loss in the HFD mouse model. Although further investigations are needed to elucidate the potential effectiveness of chalcone-based treatment in humans, the valuable natural chalcone anti-obesity potential could be considered as a useful tool for the formulation of innovative nutraceuticals for the management of this high-social-impact disease.

## Figures and Tables

**Table 1:** Molecular targets of natural chalcones with anti-obesity potential.

Chalcone	Cell Model	Dose	Mechanism	Ref.
Cardamomin	3T3-L1	10 or 30 µM	PPARγ ↓, FABP4 ↓, C/EBPα ↓, PRDM16 ↑, PGC1α ↑, UCP1 ↑, ERK ↑, (PKA)-mediated browning ↑	[31]
	3T3-L1	3, 6, 12, 25, and 50 µM	C/EBPα ↓, FABP4 ↓, LPAATθ ↓, DGAT1 ↓, SREBP1 ↓, FAS ↓	[38]
Licochalcone A	3T3-L1	5 and 10 µM	PPARγ ↓, C/EBPα ↓, SREBP1c ↓, FAS ↓, SCD1 ↓, GPAT ↓ CPT1 ↑, ACC ↓, AMPK ↑, PPAR-α ↑, UCP1 ↑,	[40]
	-	35 mg/mL	inhibition of the pancreatic lipase enzyme	[42]
Butein	3T3-L1	5, 10, and 25 μM	PPARγ ↓, C/EBPα ↓, Nrf2 ↑, HO-1 ↑	[46]
	3T3-L1	30 µM	HO-1 ↑	[52]
	C3H10T1/2 cells	10 mM	Lipid accumulation ↓, PPARγ ↓, aP2 ↓, and LPL 100%	[53]
			PPARγ, aP2, and LPL 50% ↓	
	3T3-L1	1–40 µM	AMPK ↑, PPARγ ↓, C/EBPα ↓, GPAT-1 ↓, CPT1 ↑, ACC ↑	[56]
Panduratin A	3T3-L1, HepG2, and L6 skeletal muscle cells	-	Triglyceride accumulation ↓, AMPK ↑, PPARγ ↓, C/EBPα ↓, ACC ↓, FAS ↓, SREBP1c ↓, PPARα ↑, PGC-1α ↑, CPT-1L ↑, CPT-1M ↑, UCPs ↑	[59]
Isoliquiritin	Caco-2, HepG2	100 μmol/L	Cholesterol lowering, NPC1L1 ↓, HDL catabolism ↑	[64]
	3T3-L1	100 µM	Insulin-stimulated ROS production and adipocyte differentiation ↓, superoxide generation ↓, FABP4 ↓, GLUT4 ↓, PPARγ ↓, C/EBPα ↓, PTP1B oxidation ↓, AKT phosphorylation ↓	[66]
	Human adipose-derived stem cells (hASCs)		UCP1 ↑, PRDM16 ↑, JNK ↑	[69]
Xanthohumol	3T3-L1 and primary human subcutaneous preadipocytes		CIDE-A ↑, TBX-1 ↑, UCP1 ↑, ACC ↓, HSL ↑, PGC-1α ↑	[74]
	Huh-7			
	3T3-L1	1.5 mM		[75]
Flavokawains	3T3-L1	10 µg/mL	Adipocyte differentiation ↓, phosphorylation of ERK ↑, lipid accumulation ↓, (C/EBP)-β ↓, C/EBPα ↓, PPARγ ↓	[77]
Xanthoangelol	3T3-L1	1, 5, 10, and 30 µM	C/EBP ↓, C/EBP ↓, PPARγ ↓	[78]
4-Hydroxyderricin	3T3-L1	1, 5, 10, and 30 µM	Glycerol-3-phosphate acyl transferase-1 ↓, CPT ↑	[78]

Abbreviations: PR/SET Domain 16; Carnitine palmitoyltransferase 1 (CPT1); Glycerol-3-phosphate acyl transferase (GPAT)-1; Peroxisome proliferator-activated receptor gamma (PPAR) γ; Peroxisome proliferator-activated receptor alpha (PPAR-α); fatty-acid-binding protein 4 (FABP4), and CCAAT/enhancer-binding proteins (C/EBPs) α or (C/EBPs) β; Heme oxygenase-1 (HO-1); Extracellular signal-regulated kinase (ERK); Nuclear transcription factor erythroid 2-related factor 2 (Nrf2); Protein-tyrosine phosphatase 1B (PTP1B); Uncoupling protein 1 (UCP1); Niemann Pick C1 Like 1 (NPC1L1); Acetyl-CoA carboxylase (ACC); C-Jun N-terminal kinase (JNK); PR domain containing 16 (PRDM16), Lysophosphatidic acid acyltransferase theta (LPAATθ), Diacylglycerol acyltransferase 1 (DGAT1), Sterol regulatory element-binding protein 1 (SREBP1); Sterol regulatory element-binding protein 1 isoform c (SREBP1c); Fatty acid synthase (FAS); Peroxisome proliferator-activated receptor gamma coactivator 1-alpha (PGC1α); Protein kinase A (PKA); AMP-activated protein kinase (AMPK).

## Data Availability

Not applicable.
